# The Hemostatic Net in Aesthetic Surgery: An Expert Video Roundtable Discussion

**DOI:** 10.1093/asjof/ojag086

**Published:** 2026-05-11

**Authors:** Foad Nahai, André Auersvald, Luiz A Auersvald, Thomas Gerald O’Daniel, Marc D Pacifico

The authors are pleased to present an expert roundtable discussion on hemostatic nets in plastic surgery (Video). The roundtable was recorded remotely to feature a panel of leading experts in aesthetic surgery from Brazil, the United Kingdom, and the United States. The discussion began with an explanation of the history of the hemostatic net from Dr Andre Auersvald and Dr Luiz Auersvald, who pioneered and popularized the technique. They originally conceived of the net as a solution to hematomas in face and neck procedures. Subsequent research demonstrated the hemostatic net's effectiveness in treating hematomas and its overall safety. The panelists then discussed the broader uses of the net, including skin redistribution, redraping, and redistribution of tension on the skin in breast implant surgery and other procedures. They concluded by discussing the hemostatic net's potential effects on skin pigmentation and how they manage this, with the panelists agreeing that hyperpigmentation is rare and manageable when it does occur.

